# Supplementary material for: Diversity of Bacterial Soft Rot-Causing Pectobacterium Species Affecting Cabbage in Serbia
Source: Microorganisms. 2023 Jan 29;11(2):335. doi: 10.3390/microorganisms11020335 (PMC9962274; doi:10.3390/microorganisms11020335)
Supplement: Supplementary file 1 [file microorganisms-11-00335-s001.zip › microorganisms-2120330-supplementary.pdf]

Diversity of bacterial soft rot-causing *Pectobacterium* species affecting cabbage in Serbia

Aleksandra Jelušić[1], Petar Mitrović[2], Sanja Marković[1], Renata Iličić[3], Predrag Milovanović[4], Slaviša Stanković[5] and Tatjana Popović Milovanović[6,*]

[1] University of Belgrade, Institute for Multidisciplinary Research, Kneza Višeslava 1, 11030 Belgrade, Serbia;
[2] Institute for Field and Vegetable Crops, National Institute of the Republic of Serbia, Maksima Gorkog 30, 21000 Novi Sad, Serbia;
[3] Faculty of Agriculture, University of Novi Sad, Trg Dositeja Obradovića 8, 21000 Novi Sad, Serbia;
[4] Agrosava doo, Palmira Toljatija 5, 11070 Belgrade, Serbia;
[5] University of Belgrade, Faculty of Biology, Studentski Trg 16, 11000 Belgrade, Serbia;
[6] Institute for Plant Protection and Environment, Teodora Drajzer 9, 11040 Belgrade, Serbia.
* Correspondence: tanjaizbis@gmail.com (T.P.M.); jelusic.aleksandra@gmail.com (A.J.)

SUPPLEMENTARY METERIAL

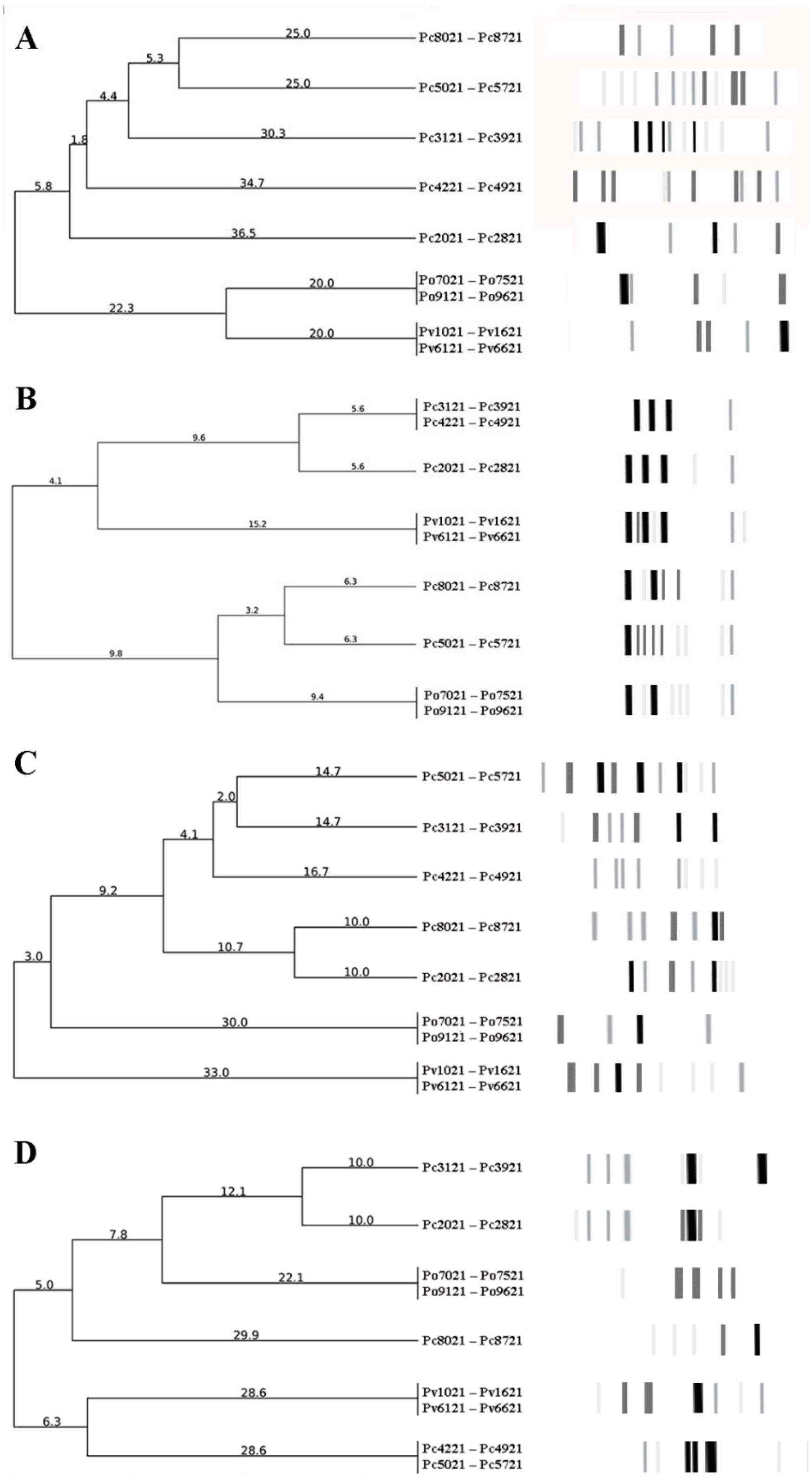

Figure S1. Unweighted pair group method with arithmetic mean (UPGMA) phylogenetic trees of the 67 tested cabbage *Pectobacterium* spp. isolates and virtual gel images depicting rep-PCR fingerprinting patterns for each of the obtained isolate groups based on (**A**) BOX-PCR, (**B**) ERIC-PCR, (**C**) GTG$_5$-PCR, and (**D**) REP-PCR.
